# Real-Time Coronary Artery Dominance Classification from Angiographic Images Using Advanced Deep Video Architectures

**DOI:** 10.3390/diagnostics15101186

**Published:** 2025-05-08

**Authors:** Hasan Ali Akyürek

**Affiliations:** Department of Biostatistics and Medical Informatics, Faculty of Medicine, Necmettin Erbakan University, Konya 42080, Türkiye; hakyurek@erbakan.edu.tr

**Keywords:** automated diagnosis, cardiovascular imaging, coronary angiography, coronary artery dominance, deep learning, MMAction2, Temporal Segment Networks (TSN), transformer models, video classification, VideoMAEv2

## Abstract

**Background/Objectives**: The automatic identification of coronary artery dominance holds critical importance for clinical decision-making in cardiovascular medicine, influencing diagnosis, treatment planning, and risk stratification. Traditional classification methods rely on the manual visual interpretation of coronary angiograms. However, current deep learning approaches typically classify right and left coronary artery angiograms separately. This study aims to develop and evaluate an integrated video-based deep learning framework for classifying coronary dominance without distinguishing between RCA and LCA angiograms. **Methods**: Three advanced video-based deep learning models—Temporal Segment Networks (TSNs), Video Swin Transformer (VST), and VideoMAEv2—were implemented using the MMAction2 framework. These models were trained and evaluated on a large dataset derived from a publicly available source. The integrated approach processes entire angiographic video sequences, eliminating the need for separate RCA and LCA identification during preprocessing. **Results**: The proposed framework demonstrated strong performance in classifying coronary dominance. The best test accuracies achieved using TSNs, Video Swin Transformer, and VideoMAEv2 were 87.86%, 92.12%, and 92.89%, respectively. Transformer-based models showed superior accuracy compared to convolution-based methods, highlighting their effectiveness in capturing spatial–temporal patterns in angiographic videos. **Conclusions**: This study introduces a unified video-based deep learning approach for coronary dominance classification, eliminating manual arterial branch separation and reducing preprocessing complexity. The results indicate that transformer-based models, particularly VideoMAEv2, offer highly accurate and clinically feasible solutions, contributing to the development of objective and automated diagnostic tools in cardiovascular imaging.

## 1. Introduction

The coronary artery system is essential for ensuring the adequate oxygenation of the heart muscle. Coronary artery dominance, one of the most significant morphological characteristics of this system, is determined based on the main coronary artery from which the posterior descending artery (PDA) originates. Anatomically, dominance is classified as right if the PDA arises from the right coronary artery (RCA), left if it originates from the left circumflex artery (LCx), and co-dominant if stemming from both arteries simultaneously [1,2].

Determining coronary dominance is clinically significant not only from an anatomical standpoint, but also for the diagnosis, grading, and therapeutic planning of cardiovascular pathologies such as coronary artery disease (CAD). The accurate classification of dominance is a fundamental prerequisite in scoring systems such as SYNTAX and Gensini [3,4,5]. However, the determination of dominance is generally performed manually through the interpretation of angiographic images, leading to considerable inter-observer variability and diagnostic uncertainty [6,7]. In this context, deep learning-based models have recently been employed to automate this classification process. The first deep learning approach based on RCA image classification was developed by Kruzhilov, et al. [8], employing ConvNeXt and Swin architectures for 2D frame-based classification to predict the source of the PDA from RCA images. However, this model can be limited by factors such as RCA occlusion, small vessel diameter, and inadequate image quality, necessitating alternative decisions using LCA images in these scenarios [8].

To overcome these limitations, the CoronaryDominance dataset Kruzhilov, et al. [9] was developed as a comprehensive video collection comprising images from RCA and LCA sources for a total of 1574 cases. Each case in the dataset is classified as either RCA or LCA images and enriched with parameters such as occlusion, artifacts, low quality, and small RCA during labeling. The process involves initial RCA occlusion detection, followed by classification based on RCA if open or LCA if occluded. This structure requires complex decision branching, involving prior data separation, prerequisite controls, and arterial branch information [9].

Given the critical role of coronary dominance classification in risk stratification, revascularization planning, and prognostic assessment, it is crucial to ensure accurate and reproducible classification [10,11]. However, the manual interpretation of angiographic images can lead to inconsistencies, particularly in borderline anatomical conditions such as co-dominance or complex cases such as small-caliber right coronary arteries. This variability may cause differences in clinical decision-making processes and biases in outcome evaluations. Therefore, there is an increasing demand for robust automated systems capable of performing coronary dominance classification with minimal dependence on subjective human interpretation.

Traditional quantitative methods rely heavily on explicit vessel segmentation and the determination of anatomical reference points, whereas deep learning approaches offer scalable and real-time solutions. Deep learning architectures are capable of learning complex spatiotemporal patterns directly from noisy or ambiguous angiographic images without requiring human input [12,13]. This capability facilitates faster and more reliable outcomes in clinical practice, significantly reducing the need for human interventions such as manual corrections. The unreliability of traditional methods is often exacerbated by the presence of low-quality angiographic images, primarily due to their dependence on manually defined features and rule-based systems [13].

The end-to-end architectures characteristic of deep learning models offer flexibility in handling variations in imaging protocols. For instance, architectures like U-Net and related designs effectively capture features across multiple scales [14,15], enabling the direct and objective processing of angiographic images and ensuring a more consistent classification process [16]. The ability to rapidly process image data is particularly advantageous in emergency settings or high-patient-volume scenarios, further positioning deep learning-based approaches as the preferred choice [17].

The transition from rule-based systems to data-driven inference frameworks, supported by deep learning methods, has significantly advanced medical image segmentation [18]. Deep learning enhances the automated segmentation process by comprehensively understanding the medical image content, thus conserving time and effort [19]. Notably, several studies have demonstrated that deep learning techniques achieve high diagnostic accuracy and produce stable predictions [20]. With these strengths, deep learning continues to open up new horizons for the effective processing and analysis of angiographic imaging.

Distinctively, a system that is capable of directly classifying coronary dominance regardless of arterial origin without distinguishing whether the image originates from RCA or LCA is proposed in this study. For instance, the single-step decision-making process allows the model to process all angiographic video sequences within a unified system without prior knowledge of arterial branches or the need for separate model training. Thus, there is no necessity for a separate arterial classifier or preliminary conditions. Similarly, the approach provides greater tolerance to complex anatomies, especially in cases where the RCA is hypoplastic, occluded, or where PDA originates from unusual locations. Rather than determining the image sources separately, the decision is made holistically, thereby increasing the model’s robustness against domain shifts and anatomical variations. Additionally, from a clinical integration standpoint, this approach enables streamlined incorporation into real-time systems without additional software to distinguish LCA and RCA images, facilitating direct classification from angiographic image streams and resulting in practical utility requiring minimal intervention [8,21,22,23].

The architectures supporting this approach include Temporal Segment Networks (TSNs), which make up a Convolutional Neural Network (CNN)-based architecture extracting long-term motion patterns through sparse temporal sampling [24,25]. Video Swin Transformer, which is a transformer architecture analyzing images through shifted-window-based local–global attention blocks, is capable of finely discriminating clinically relevant details [26]. VideoMAEv2, which is a next-generation masked autoencoder architecture that learns video representations in a self-supervised manner, significantly reduces dependency on labeled data [27]. These three architectures were trained to classify video sequences regardless of arterial origin, with detailed comparative evaluations of their performances. Assessments conducted from the perspectives of clinical consistency, sensitivity to class imbalance, domain shift tolerance, and interpretability demonstrated the superiority of the models, not only in terms of accuracy but also in their real-world applicability.

### Literature Review

In recent years, artificial intelligence (AI)-based methods have offered revolutionary advancements in the image-based diagnosis of cardiovascular diseases. Ferdowsi et al. (2025) comprehensively reviewed the current applications of AI in the diagnosis, prediction, and classification of coronary heart disease, specifically elaborating on how deep-learning approaches improve diagnostic accuracy and risk classification across imaging modalities such as angiography, CT, and echocardiography [28].

Similarly, Khera et al. (2024) [29] emphasized the transformative potential of artificial intelligence (AI) across all aspects of cardiovascular practice and research. Their comprehensive review highlighted that the rapid advancements in AI-driven technology are opening new frontiers in cardiovascular care, encompassing innovations such as novel diagnostic modalities, digital native biomarkers, and high-performance tools for evaluating quality of care and prognosticating clinical outcomes. These innovations are particularly valuable for extending cardiovascular screening and monitoring capabilities, notably benefiting populations historically lacking access to specialized care. Moreover, Khera et al. (2024) [29] underscored that AI is accelerating biological and clinical discoveries, paving the way towards more personalized, precise, and effective cardiovascular care. They envisioned a future where multimodal cardiovascular AI systems would be seamlessly integrated at the point of care, significantly reshaping diagnostic and therapeutic practices. Finally, they defined a critical roadmap, advocating for equitable and regulated adoption strategies to ensure fairness, safety, and effective partnerships, thereby enabling optimal cardiovascular health outcomes for society at large [29].

Additionally, Akgül et al. (2024) [30] presented remarkable results using their AI-based system designed for the real-time detection of stenosis in coronary angiographic images. They emphasized the exceptional capability of their approach, achieving notably high performance in terms of both F1-score and mAP@50 metrics. Their findings clearly illustrate the considerable potential of object detection-based AI models to enable the automatic and precise quantification of stenosis severity within clinical settings [30]. These recent reviews and applications underline that AI is becoming an increasingly central tool in cardiovascular imaging, thereby providing a solid foundation for the innovative contribution of this study in this field.

Deep learning-based video architectures such as Temporal Segment Networks (TSNs), Video Swin Transformer, and VideoMAEv2 have gained widespread use in medical video imaging domains, including cardiac imaging, endoscopy, and surgical video analysis. These approaches have demonstrated promising results in tasks such as classification, segmentation, and anomaly detection, highlighting their substantial potential for capturing complex spatiotemporal patterns within medical data.

Zeng, et al. [31] developed a hybrid architecture called Video-TransUNet, integrating temporal feature fusion and transformer modules to achieve instance segmentation in CT video sequences. Their model successfully segmented anatomical structures such as the bolus and pharynx/larynx, thus illustrating the flexibility of video-based models in analyzing dynamic, high-resolution medical images. Similarly, Wang, et al. [32] proposed a deep learning approach that leverages temporal–spatial information for coronary artery segmentation from angiographic sequences.

The capabilities of the VideoMAEv2 architecture were demonstrated by Tian, et al. [33] in a study focused on action recognition in endoscopic surgical videos. They enhanced model performance through temporal data augmentation and hybrid pre-training datasets. While their research specifically targeted surgical video analysis, the methods employed suggest broader potential for VideoMAEv2 applications in medical video analysis.

Despite these advancements, a significant gap remains in the literature regarding the direct application of video-based deep learning models specifically for coronary angiography or coronary artery dominance classification. Although video-based architectures have proven successful for tasks like stenosis detection or vessel segmentation in cardiovascular imaging, studies explicitly targeting coronary dominance classification are notably scarce. This gap might stem from clinical challenges such as the complexity of coronary anatomy, the presence of imaging artifacts, and the precision required for distinguishing between right and left dominance.

This gap has considerable clinical relevance. The accurate determination of coronary artery dominance is critical in preoperative assessments, surgical planning, and widely used scoring systems such as SYNTAX and Gensini. Integrating advanced video-based deep learning models into angiographic classification tasks could enhance diagnostic accuracy and operational efficiency, potentially leading to improved patient outcomes. The existing literature demonstrates that architectures such as TSNs, Video Swin Transformer, and VideoMAEv2 can be effectively integrated into medical video analysis. However, their application to specialized clinical tasks such as coronary dominance classification remains insufficiently explored. This study addresses this gap by proposing a source-independent, automated dominance classification system for angiographic video data, thereby making a significant technical and clinical contribution to the literature.

## 2. Materials and Methods

### 2.1. Dataset Description

The dataset utilized in this study is based on the CoronaryDominance dataset initially published by Kruzhilov et al. [9], recognized as the most comprehensive coronary dominance dataset available in the literature. This dataset comprises fully labeled angiographic video sequences containing separate projections of the right coronary artery (RCA) and left coronary artery (LCA). Each video sequence is labeled as either right dominance (RD) or left dominance (LD) according to the arterial origin of the posterior descending artery (PDA). However, in line with the aim of this study, all video sequences were analyzed within a unified classification framework regardless of arterial branches [9].

The dataset consists of a total of 5157 angiographic video samples, of which 3584 (69.5%) are right-dominant and 1573 (30.5%) are left-dominant. This distribution closely aligns with the prevalence rates encountered in clinical practice [34,35]. The videos were obtained from 1574 unique patients, among whom 567 were diagnosed with acute coronary syndrome (ACS) and 1007 had stable coronary artery disease (CAD) [9]. Class imbalance presents a significant challenge, particularly affecting the training and validation processes for less represented classes such as left dominance, and therefore necessitates specialized strategies during model development [36].

To ensure the generalization capability of the models during training and evaluation, the dataset was systematically divided into three subsets: 70% training, 15% validation, and 15% testing, with each subset reflecting the class distribution of the full dataset. Details of the subsets are provided in Table 1.

However, the dataset distribution accurately reflects real-world clinical prevalence, as also reported in multiple cardiology studies [e.g., SYNTAX, Gensini]. In this context, the principle that “Training data needs to reflect the real world” was followed [37]. Over-sampling or synthetic balancing was deliberately not applied, as it could compromise the real-world applicability of the model. In order to ensure fairness, stratified data splitting was performed, and the performance of the model was assessed using class-sensitive evaluation metrics, including recall, F1-score, and the Matthews Correlation Coefficient (MCC), in addition to overall accuracy.

Unlike the conventional approaches, this study did not require the separate treatment of RCA and LCA images. Instead, all video sequences were processed in a unified manner without considering the arterial origin, significantly reducing the structural complexity in the model’s decision-making process. This approach not only simplifies the data processing pipeline, but also potentially enables the model to capture a broader spectrum of anatomical variations, enhancing generalizability [38,39].

The primary advantages of employing a unified RCA–LCA video data structure include increased data volume, enhanced anatomical diversity, reduced bias, a simplified model architecture, and minimized labeling inconsistencies. Potential disadvantages, however, may include the loss of anatomical specificity, decreased interpretability, and the risk of masking specific arterial anomalies [40,41].

Additionally, the influence of demographic and clinical factors on classification accuracy was also considered. Factors such as age, gender, associated comorbidities, angiography equipment, and regional anatomical variations are known to significantly affect model performance [42,43].

### 2.2. Data Preprocessing

A comprehensive data preprocessing pipeline was designed to enhance the accuracy and generalizability of deep-learning models for coronary dominance classification. The applied processes standardized the image quality and efficiently represented the temporal characteristics of video data in the models.

Initially, all angiographic frames were resized to a resolution of 224 × 224 pixels. This consistent resizing eliminated resolution variations across different video sources, enabling a uniform input into the neural networks. Images were normalized using mean and standard deviation values from the ImageNet dataset, facilitating the effective use of pretrained models during transfer learning and fine-tuning processes, and ensuring more stable gradient flow throughout the training.

Given the inherently time-dependent nature of angiographic video sequences, temporal sampling formed a crucial component of the preprocessing pipeline. Each video sequence was sampled at regular intervals based on its total frame count, with frames selected according to the structural requirements of each model. Particularly for the Temporal Segment Network (TSN) architecture, a sparse temporal sampling strategy evenly spaced throughout the video was employed, following the original method [25]. Similarly, the Video Swin Transformer and VideoMAEv2 architectures were structured to follow the temporal sampling protocols appropriate to their respective training strategies [27].

The preprocessing approach adopted in this study fundamentally differs from traditional methods of coronary dominance classification. Typically, RCA and LCA projections are separately identified and individually input into models after an arterial branch classification stage, necessitating branched decision structures [9]. However, for the approach in this study, no preliminary information about arterial origin was introduced into the decision-making process. Hence, the model directly analyzed the entire video sequence without knowledge of arterial branches for coronary dominance classification.

This methodological simplification reduced the complexity of the model architecture and enabled the models to autonomously learn artery-specific variations internally. Consequently, the developed system not only presented a simpler and more efficient structure, but also established a more flexible, generalizable, and clinically integrable AI-based decision support system.

### 2.3. Model Implementations

In this study, three distinct video-based deep learning architectures were utilized for the automatic classification of coronary dominance from angiographic video sequences: Temporal Segment Networks (TSNs), Video Swin Transformer, and VideoMAEv2. Each model was carefully selected due to its capacity for learning spatiotemporal data patterns and its potential applicability in clinical imaging.

#### 2.3.1. Temporal Segment Networks (TSNs)

Temporal Segment Networks (TSNs) comprise a deep learning architecture specifically developed to efficiently learn temporal patterns from long-duration video sequences, and this architecture has increasingly been adopted in medical video analysis applications. Traditional video classification methods typically rely on densely sampling consecutive frames, resulting in high computational loads and prolonged processing times. To address this issue, TSNs employ a sparse sampling strategy by dividing each video sequence into a fixed number of segments, randomly selecting one representative frame from each segment. These sampled frames are independently processed by Convolutional Neural Networks (CNNs) and subsequently fused using temporal average pooling to perform video-level classification [25].

**Figure 1:**
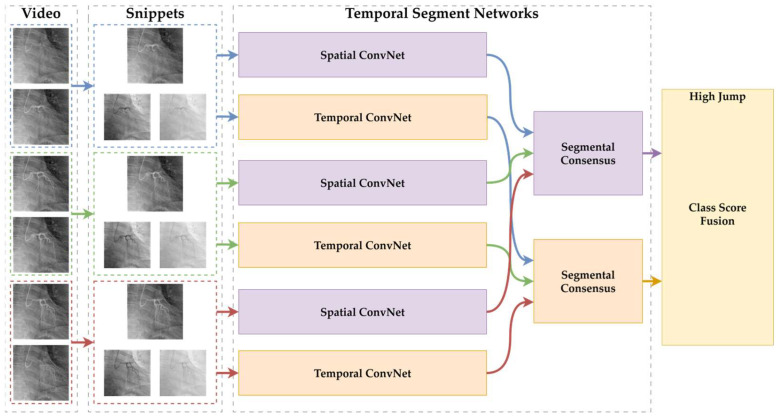
Overview of the Temporal Segment Network (TSN) framework for video-based action recognition inspired by [25]. Red, Green and Blue colors represent different clips taken from the video sequence.

The general architecture of the Temporal Segment Networks (TSNs) used in this study is illustrated in Figure 1. The diagram depicts the input video sequences being divided into temporal segments, from which sparse frames are sampled. These sampled frames are individually processed by Convolutional Neural Networks (CNNs), and the final class predictions are subsequently generated through a segmental consensus mechanism that aggregates the individual segment outputs.

In TSNs, the input video V is divided into K equal-length temporal segments, as shown in Equation (1).

(1) $$V=\left\{S_{1},S_{2},\ldots ,S_{K}\right\}$$

From each segment $S_{k}$, a short snippet $T_{k}$ is sampled. Each snippet is processed by a shared convolutional backbone $f(T_{k};\;\theta )$, producing prediction logits. A segmental consensus function G(⋅) aggregates the logits, and softmax is applied to produce the final prediction $\hat{y}$, as in Equation (2).

(2) $$\hat{y}=Softmax\left(G\left(f\left(T_{1};\theta \right),f\left(T_{2};\;\theta \right),\ldots ,f\left(T_{K};\theta \right)\right)\right)$$

Here, θ denotes the shared network weights. In this study, K=3, and average pooling was used as the consensus function G. This formulation allows for the modeling of long-term temporal information across angiographic video sequences.

This structure is particularly effective for images such as coronary angiographic videos, which are characterized by long durations, high resolutions, and clinically significant temporal information. By sampling frames across segments, the model captures slowly evolving but diagnostically crucial dynamics over time. For example, subtle temporal variations reflecting coronary artery perfusion patterns—such as contrast distribution—can be effectively modeled within the TSN architecture. Furthermore, its sparse sampling significantly reduces both the training and inference costs, enhancing the model’s potential for real-time clinical implementation [24,25].

The segment-based approach also confers robustness against variations in patient anatomy, imaging techniques, and motion artifacts. Consequently, the TSN offers substantial generalization capabilities, making it adaptable to diverse clinical scenarios. However, the TSN architecture also has inherent limitations. It does not account for the sequential context between frames across segments, potentially resulting in a loss of temporal information such as event timing. Additionally, rapid-onset and short-duration clinical events appearing in only a few segments might be overlooked by the model. Given that the model performance heavily depends on the representational quality of the selected frames, any sampling inaccuracies may significantly impair the classification accuracy [24,25].

The strengths and limitations of TSNs become more apparent when compared to more recent video analysis architectures. For example, transformer-based models like the Video Swin Transformer leverage attention mechanisms to more effectively model sequential temporal contexts and uncover detailed relationships. However, these architectures are considerably more complex, computationally demanding, and typically require more labeled training data compared to TSNs. Consequently, the TSN offers a balanced solution between high efficiency and sufficient accuracy, which is especially advantageous in clinical contexts where labeled data availability and computational resources are limited [24,25].

Temporal Segment Networks (TSNs) provide an efficient, effective, and generalizable architecture for the analysis of medical video data, such as coronary angiography. In this study, TSNs served as one of the foundational comparative models for artery-independent coronary dominance classification, trained on a unified video dataset without distinguishing between RCA and LCA video sequences [32,44].

**Figure 2:**
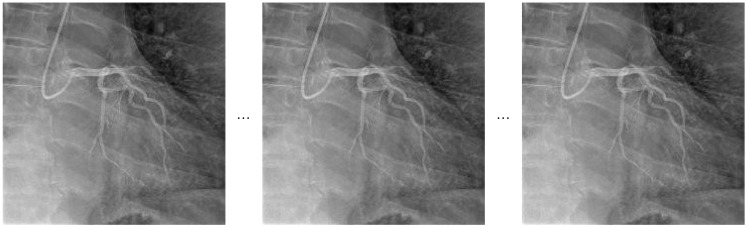
Representative frame sampling for Temporal Segment Network.

The temporal sampling strategy adopted within the TSN architecture involves dividing each video sequence into evenly spaced segments and selecting representative frames from each segment. A visual illustration of this approach is provided in Figure 2.

#### 2.3.2. Video Swin Transformer

The Video Swin Transformer (VST) is an innovative, attention-based architecture specifically developed to model complex spatiotemporal relationships within video data. Unlike traditional Convolutional Neural Networks, the VST employs a hierarchical structure and shifted-window mechanisms to process both spatial and temporal information at multiple scales [26,45]. Due to these characteristics, the model is particularly well suited for medical imagery, including coronary angiographic videos, which often involve high-resolution and dynamically evolving structures.

**Figure 3:**
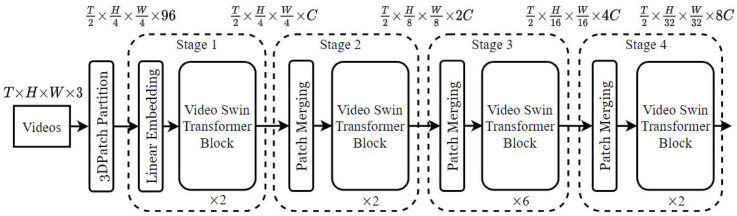
Block diagram of the Video Swin Transformer for video-based action recognition [26].

The structural components of the Video Swin Transformer, along with its hierarchical attention mechanism and shifted window operations, are depicted in Figure 3 as a block diagram tailored for video-based action recognition applications.

The Video Swin Transformer partitions an input video clip into non-overlapping 3D windows of sizes (P, M, and M) along the time, height, and width axes. Each window is processed using local self-attention. Within each window, self-attention is computed as in Equation (3).

(3) $$Attention\left(Q,K,V\right)=Softmax\left(\frac{QK^{T}}{\sqrt{d}}+B\right)*V$$

where

- Q, K, and $V\in R^{n\;x\;d}$ are the query, key, and value matrices derived from the patch embeddings;
- d is the attention dimension;
- B is the relative position bias matrix.

This architecture applies shifted windows between layers to enable cross-window interaction, allowing for the hierarchical capture of spatiotemporal patterns, which is particularly beneficial for interpreting the flow and anatomical continuity in coronary angiograms.

The hierarchical architecture of the VST enables analysis at various resolution levels, making it possible to simultaneously evaluate the overall anatomical patterns of vessels and micro-level variations. This multi-scale learning capability offers substantial potential for accurately distinguishing between normal and pathological vessel segments [46]. In clinical scenarios such as coronary angiography, where detailed vascular analysis is crucial, this structural design transforms the VST into a powerful diagnostic tool.

Another key component of the VST is its shifted-window mechanism, designed to overcome the high computational demands typically associated with conventional global self-attention structures. In this approach, attention computation is limited to local windows at each step, followed by systematically shifting window positions to facilitate information exchange between neighboring regions [47]. This approach ensures that detailed local information is captured while maintaining global contextual coherence. The identification of clinically significant details such as minor calibration differences, vascular stenosis, or variations in contrast intensity in angiographic videos is therefore handled more precisely through this local attention structure [47].

The computational efficiency of the VST is particularly notable. Restricting attention to local windows enables the real-time analysis of high-resolution video sequences. Moreover, the model is highly robust against resolution variability, ensuring consistent performance despite fluctuations in the image quality arising from different imaging devices or settings [46]. By effectively contextualizing physiological variations or pathological developments across long sequences, the VST can fulfill a robust classifier role in clinical applications.

Nevertheless, certain limitations of the Video Swin Transformer must also be acknowledged. The multi-layered architecture and window-based attention computations introduce significant complexity into the training and optimization processes, requiring meticulous hyperparameter tuning [48]. Additionally, as with transformer-based models generally, a lack of inherent interpretability remains a concern. The transparency of decision-making processes is critically important for clinical trust and acceptance [49].

Model performance is strongly dependent on data quality. In contrast to self-supervised methods like VideoMAEv2, the VST relies on supervised learning paradigms and thus requires high-quality, balanced datasets. Model performance may particularly suffer in clinical scenarios that are underrepresented [50]. Moreover, the dependence of the attention mechanism on window size and positioning may lead to an incomplete representation of rapidly occurring temporal events [51].

Overall, the Video Swin Transformer presents a robust alternative for coronary dominance classification due to its advanced architecture that effectively integrates spatiotemporal pattern modeling. Its high accuracy potential, resolution tolerance, and contextual information extraction capabilities significantly facilitate integration into clinical decision support systems. However, the careful management of challenges such as interpretability, training complexity, and data sensitivity remains essential to enhance the clinical validity and practical adoption of this architecture [52].

**Figure 4:**
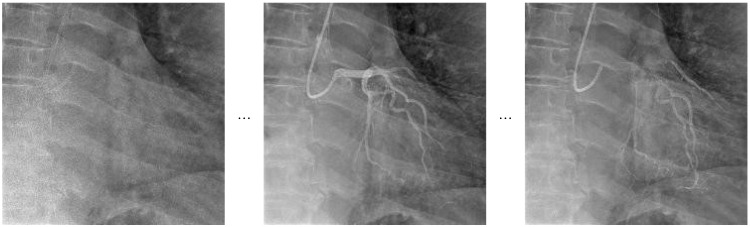
Representative frame sampling for Video Swin Transformer.

The frame sampling strategy utilized in the Video Swin Transformer, which focuses on extracting consecutive frames to effectively capture detailed temporal dynamics, is presented in Figure 4 as a visual representation.

#### 2.3.3. VideoMAEv2

VideoMAEv2 is a masked autoencoder (MAE)-based model designed to learn powerful representations from video data in an unsupervised manner. This architecture efficiently reduces redundancy within video sequences, utilizing a dual-masking strategy to simultaneously capture the spatial–temporal context [27]. A crucial feature of VideoMAEv2 is its ability to derive meaningful representations during pre-training from large volumes of unlabeled medical video data, significantly enhancing performance in subsequent supervised classification tasks [27].

**Figure 5:**
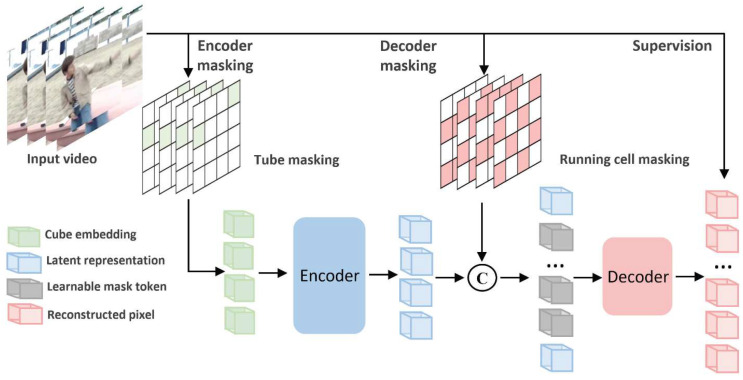
Overview of the VideoMAEv2 for video-based action recognition [27].

The overall architecture and operational concept of the VideoMAEv2 model, which is built upon a masked autoencoding framework and designed to learn spatiotemporal representations from video inputs, is depicted in Figure 5 for the task of video-based action recognition.

VideoMAEv2 is a masked video autoencoder framework that learns spatiotemporal representations via self-supervised pretraining. Given an input video x, a large portion of the tokens are masked. The encoder processes only the visible subset $x_{vis}$, as in Equation (4).

(4) $$z=Encoder(x_{vis})$$

The decoder reconstructs the masked patches from latent representation *z* and placeholder tokens, as in Equation (5).

(5) $${\hat{x}}_{masked}=Decoder(z,[MASK])$$

The pretraining loss is computed as in Equation (6):

(6) $$L_{MAE}=\frac{1}{\left|M\right|}*\sum_{i\in M}{\left\|x_{i}-{\hat{x}}_{i}\right\|}^{2}$$

where M is the set of masked patch indices.

After pretraining, the encoder is fine-tuned using a classification head for coronary dominance prediction. This design improves the robustness against artifacts and incomplete views by learning to reconstruct vessel patterns from limited visual information.

The masked autoencoding approach is particularly effective in video-based medical imaging applications such as coronary angiography. The MAE structure masks a substantial portion of input data, striving to reconstruct a full representation of the original video from the remaining small visible parts. Consequently, the model learns to notice not only prominent anatomical structures, but also contextually critical regions that provide limited visual cues. VideoMAEv2 further enhances this methodology by applying masking to both the encoder and decoder components, facilitating a bi-directional learning flow [27].

Within this architecture, “tube masking” and “running cell masking” methods enable integrated spatial and temporal assessments. This capability allows for the effective analysis of subtle variations in vascular structures, delicate changes in blood flow dynamics, and inter-segment contextual relationships. The reconstruction-based training strategy of VideoMAEv2 indirectly identifies which portions of the video are most influential in decision-making processes, thereby improving the interpretability of the model [53,54].

One of the primary advantages of VideoMAEv2 is its capacity for efficient learning from extensive yet unlabeled video collections. This ability presents a substantial solution to the commonly encountered problem of limited labeled data in the healthcare domain. Additionally, the high scalability of the model facilitates adaptation across various clinical scenarios and enhances the integration potential into real-time systems due to its computational efficiency [55,56].

Nevertheless, VideoMAEv2 faces certain challenges. The sophisticated masking strategies employed by the model necessitate careful hyperparameter tuning and systematic data preparation procedures. Additionally, the reconstruction-based learning paradigm demands high-quality, consistent data for effective training. Artifacts in images or sudden variations in clinical scenarios may diminish learning effectiveness. However, the model’s intrinsic capability to reconstruct partially corrupted or incomplete data confers significant advantages in overcoming these limitations [57].

**Figure 6:**
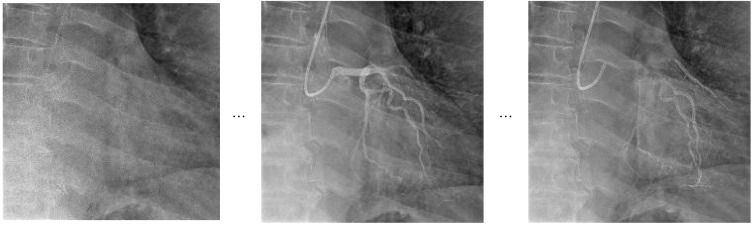
Representative frame sampling for Video Masked AutoEncoder V2.

The frame sampling technique utilized in the Video Masked AutoEncoder V2 model, which selects temporally distributed frame segments to facilitate the reconstruction of masked video inputs, is visually presented in Figure 6.

Overall, VideoMAEv2 presents a deep and balanced approach for extracting spatiotemporal features, demonstrating substantial potential to yield robust, accurate, and interpretable results for complex classification tasks such as coronary artery dominance. Its ability to reduce dependency on labeled data, coupled with its high precision in identifying pathological variations within fine vascular structures, makes it a highly suitable candidate for advanced medical imaging systems.

### 2.4. Experimental Setup and Evaluation Metrics

In this study, the training and evaluation processes for all developed models were performed using the MMACTION2 (OpenMMLab) video processing framework, built upon the PyTorch (v2.5.1) deep learning library. MMACTION2 is a robust and modular open-source framework optimized for video classification tasks, enabling straightforward comparative analyses of diverse model architectures.

The experiments were conducted on a hardware platform offering a high computational capacity, specifically an Intel Core i9-14900K CPU, 64 GB DDR5 RAM, and an PNY NVIDIA RTX A4000 GPU (PNY Technologies, Parsippany, NJ, USA) with 16 GB GDDR6 memory and CUDA 11.8 support. This powerful configuration facilitated efficient model training and allowed for the evaluation of time-sensitive metrics such as inference speed.

The hyperparameters employed in model training were optimized based on preliminary experiments and recommendations from the existing literature. The AdamW optimization algorithm was utilized with an initial learning rate set at 1 × 10^−4^. A cosine decay learning rate scheduler was chosen to smoothly decrease the learning rate over epochs, promoting stable convergence during training. A mini-batch size of 32 was uniformly maintained across all models. Training was conducted for a maximum of 50 epochs, and the model performance was evaluated using accuracy as the key metric. The input dimensions were standardized at 224 × 224 pixels.

A stratified train/validation/test split (70/15/15) was adopted due to the high computational cost and memory demands associated with k-fold cross-validation in video-based transformer models. This static split ensures balanced class representation, facilitates reproducibility, and significantly reduces the training time and GPU load, making it more practical for real-world video-based deep learning scenarios.

The essential architectural characteristics and input configuration parameters of the three deep learning models evaluated in this study are presented in Table 2. The table includes details such as the model type (2D or 3D recognizer), backbone architecture, input tensor format, clip length, and frame interval, which collectively describe the structural and temporal aspects of each model’s video input processing pipeline.

The training and testing configurations for each model architecture—including the optimizer selection, learning rate, weight decay, learning rate scheduling strategy, pretrained weights, and the number of test clips—are outlined in Table 3. These parameters were carefully determined based on established practices in the literature and refined through empirical adjustments to ensure both optimal model performance and methodological consistency across all experimental comparisons.

Throughout the training process, the performance of each model was comprehensively evaluated using widely accepted classification metrics such as accuracy (%), F1-score, precision, recall (sensitivity), and inference time (ms/frame).

These metrics were selected to evaluate not only the models’ classification accuracy, but also their balance, generalizability, and computational efficiency. The inclusion of metrics sensitive to class imbalance, such as the F1-score and recall, allowed for an objective assessment of performance given the natural prevalence differences between right and left coronary dominance.

Inference time served as a critical metric for assessing the models’ feasibility for real-time clinical applications, directly influencing the practicality of performing time-sensitive analyses on angiographic video sequences.

Thus, the training protocol described here was carefully structured to ensure fair, systematic, and consistent comparisons across models, supporting the development of clinically applicable artificial intelligence solutions.

## 3. Results

### 3.1. Comparative Analysis of Results

The three deep-learning architectures utilized in this study—Temporal Segment Networks (TSNs), Video Swin Transformer, and VideoMAEv2—were trained under similar conditions and evaluated using multidimensional performance metrics. This comparative assessment considered not only basic metrics such as accuracy, but also important clinical factors, including generalization capacity, sensitivity to class imbalance, inference speed, and overall clinical applicability.

A comprehensive comparison of the classification performance metrics for the TSN, Video Swin Transformer, and VideoMAEv2 is provided in Table 4. In addition to standard metrics such as accuracy, precision, recall, and F1-score, the table also includes clinically relevant indicators such as true positive rate (TPR), false negative rate (FNR), and Matthews Correlation Coefficient (MCC), enabling a multidimensional evaluation of each model’s effectiveness in detecting coronary artery dominance patterns.

VideoMAEv2 achieved the highest overall accuracy (93.80%) and recall (90.52%), indicating its robustness in identifying both right- and left-dominant cases. It also attained a high MCC score (0.8530), reflecting a well-balanced classification performance even under class imbalance. In contrast, while the TSN achieved comparable recall (90.45%) and showed high sensitivity toward the majority class, its lower precision (76.27%) and F1-score (82.76%) suggest a higher rate of false positives compared to VideoMAEv2. Additionally, the TSN exhibited a relatively higher FPR (9.74%) and FDR (23.73%), indicating less specificity in distinguishing non-target instances.

The Video Swin Transformer demonstrated the highest precision (94.07%) and lowest FDR (5.93%), showcasing its strength in minimizing false positive predictions. Its TNR (97.29%) and FPR (2.71%) further confirmed its strong specificity. However, its slightly lower recall (86.05%) than VideoMAEv2 implies a minor limitation in detecting all true positive cases, which is critical when the minority class (e.g., left-dominant) is of clinical significance.

The inference time metrics provide crucial insights into the models’ practicality for real-time clinical use. The TSN delivered the fastest inference at 9.7 ms/frame, with VideoMAEv2 at approximately 12.3 ms/frame and the Video Swin Transformer at 14.1 ms/frame. These values indicate the clinical integration feasibility of all models, albeit suggesting the need for scenario-specific optimization balancing speed and accuracy. The inference times are shown in Table 5.

Furthermore, accuracy–efficiency curves were analyzed, revealing that a hybrid approach using the Video Swin Transformer and VideoMAEv2 could be optimal for clinical scenarios requiring both speed and reliability. Particularly, VideoMAEv2’s capability to maintain high performance even with limited labeled data makes it attractive for resource-constrained systems.

In summary, each model exhibited distinct advantages and limitations: the TSN provides practical solutions due to its low computational cost and rapid inference speed, the Video Swin Transformer excels in accuracy and contextual interpretability, and VideoMAEv2 offers balanced performance regarding data flexibility, overall accuracy, and generalization potential. These findings demonstrate the effectiveness of video-based deep-learning models in coronary dominance classification, highlighting the importance of careful model selection tailored to specific clinical requirements.

### 3.2. Main Dataset Results

After completing an extensive training and evaluation procedure on the dataset, the models were comparatively analyzed. The training, validation, and test accuracy rates of the three deep learning models are presented in Table 6. All models demonstrated high training performance, with the TSN reaching the highest training accuracy at 97.70%, followed by VideoMAEv2 at 96.65%, and the Video Swin Transformer at 95.21%. However, training accuracy alone does not reflect a model’s ability to generalize. This capability is better assessed through validation and test performances. In this regard, VideoMAEv2 achieved the highest validation (93.80%) and test accuracy (92.89%), indicating its superior consistency and robustness across different data partitions.

The area under the receiver operating characteristic curve (AUC) values for the training, validation, and test phases of all three models are summarized in Table 7. These metrics provide a robust assessment of each model’s discriminative capability, particularly in the presence of class imbalances. All models achieved excellent training AUC scores, with the TSN reaching the highest value at 0.9971, closely followed by VideoMAEv2 (0.9936) and the Video Swin Transformer (0.9836), indicating strong learning performance on the training data. However, the validation and test AUC values offer deeper insights into each model’s generalization capacity. In this regard, VideoMAEv2 stood out with the highest validation (0.9834) and test AUC (0.9781), reflecting its superior ability to distinguish between classes on unseen data.

**Figure 7:**
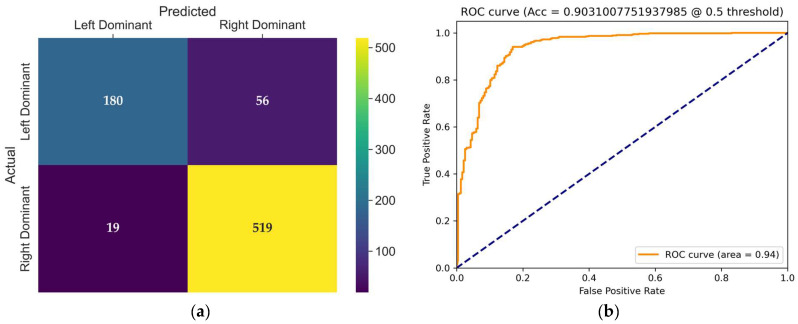
(**a**) Confusion matrix of the TSN model for coronary dominance classification; (**b**) ROC curve of the evaluated TSN model. The dashed line represents the ROC curve expected from random guess.

The confusion matrix of the TSN model, presented in Figure 7a, provides detailed insight into its classification behavior on the test set. The TSN model produced 56 incorrect classifications in the left dominance category, resulting in only 180 correct classifications. This significantly reduces the sensitivity (recall) for the left-dominant class. For the right-dominant class, the TSN model displayed strong performance, with 519 correct classifications and only 19 errors. Clearly, the model shows sensitivity to class imbalance, negatively affecting its performance in the minority (left-dominant) class.

The receiver operating characteristic (ROC) curve shown in Figure 7b illustrates the trade-off between the true positive rate and false positive rate for the evaluated model at a threshold of 0.5. The area under the curve (AUC) is 0.94, indicating excellent classification performance and strong discriminative ability in distinguishing coronary dominance classes.

**Figure 8:**
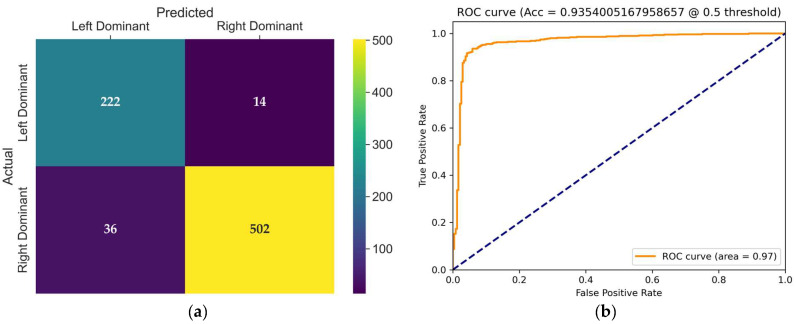
(**a**) Confusion matrix of the VST model for coronary dominance classification; (**b**) ROC curve of the evaluated VST model. The dashed line represents the ROC curve expected from random guess.

The confusion matrix of the VST model, presented in Figure 8a, provides detailed insight into its classification behavior on the test set. This model achieved balanced results across both classes. Only 14 misclassifications occurred in the left dominance class, with 36 errors in the right dominance class. Notably, the high specificity (97.29%) and low false positive rate (2.7%) illustrate stable and precise classifications. However, the recall (86.05%) indicates potential for improvement, particularly for the left dominance class.

The receiver operating characteristic (ROC) curve of the Video Swin Transformer model is shown in Figure 8b. The model achieved an AUC of 0.97 at a classification threshold of 0.5, demonstrating a strong ability to distinguish between left and right coronary artery dominance classes with high sensitivity and specificity.

**Figure 9:**
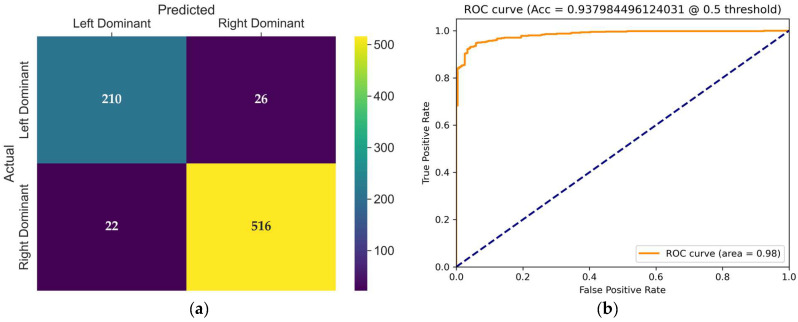
(**a**) Confusion matrix of the VideoMAEv2 model for coronary dominance classification; (**b**) ROC curve of the evaluated VideoMAEv2 model. The dashed line represents the ROC curve expected from random guess.

The confusion matrix of the VideoMAEv2 model, presented in Figure 9a, provides detailed insight into its classification behavior on the test set. VideoMAEv2 effectively balanced performance across both classes. The left dominance class achieved 210 correct classifications with just 26 errors, whereas the right dominance class achieved an impressive 516 correct classifications with 22 errors. This balanced distribution strongly positions VideoMAEv2 as a robust candidate regarding class balance, generalizability, and noise tolerance.

The receiver operating characteristic (ROC) curve shown in Figure 9b illustrates the trade-off between the true positive rate and false positive rate for the evaluated model at a threshold of 0.5. The area under the curve (AUC) is 0.98, and with a high true positive rate across almost all false positive rates, the model demonstrates excellent discriminative power and robustness in distinguishing between coronary dominance classes.

**Figure 10:**
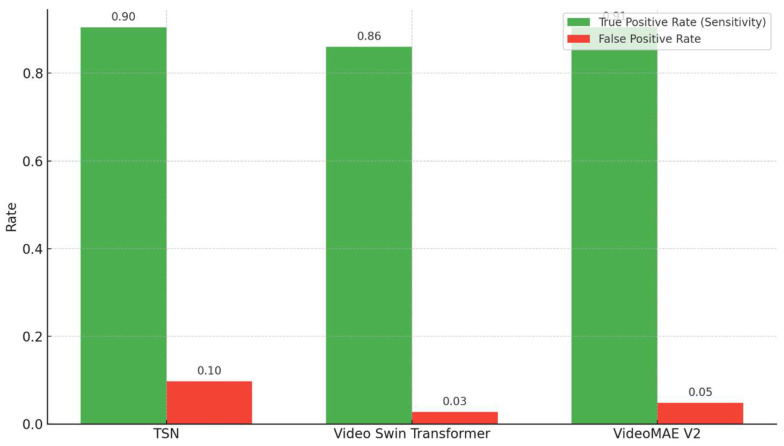
Comparison of true positive rate (sensitivity) and false positive rate across TSN, Video Swin Transformer, and VideoMAEv2 models.

As shown in Figure 10, the comparison of the true positive rate (sensitivity) and false positive rate reveals distinct performance characteristics across the three models. While the TSN achieved a relatively high sensitivity (0.90), it exhibited the highest false positive rate (0.10). In contrast, the Video Swin Transformer demonstrated a balanced trade-off, achieving lower sensitivity (0.86), but with a substantially reduced false positive rate (0.03). VideoMAEv2 offered the most favorable combination, attaining both high sensitivity (0.91) and a relatively low false positive rate (0.05), indicating its superior detection capability with minimal overdiagnosis.

**Figure 11:**
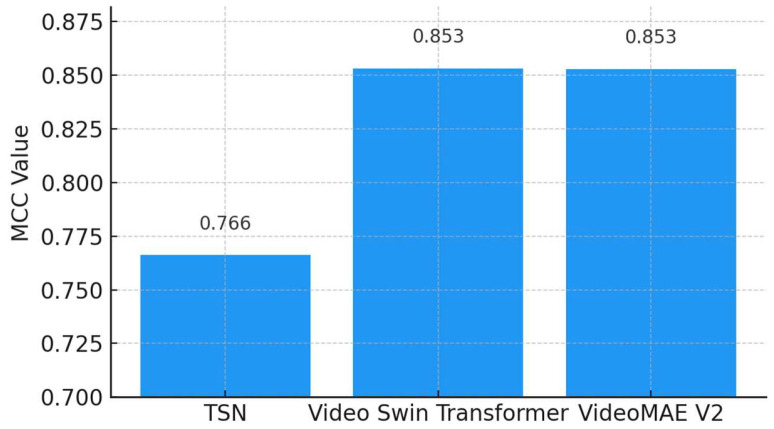
Comparison of Matthews Correlation Coefficient (MCC) among TSN, Video Swin Transformer, and VideoMAEv2.

As depicted in Figure 11, both the Video Swin Transformer (0.8533) and VideoMAEv2 (0.8530) demonstrate similarly high MCC scores, suggesting robust and balanced classification performance. The TSN shows a lower MCC value (0.7662), indicating weaker performance in balanced classification scenarios. Since MCC is a gold-standard metric for assessing balanced and reliable classification performance, these results confirm that VideoMAEv2 and the Video Swin Transformer hold statistical superiority and reliability.

### 3.3. Real Distribution Dataset Results

The evaluation of the TSN, Video Swin Transformer, and VideoMAEv2 models on the real distribution dataset provides critical insights into their generalizability within real-world clinical settings. Unlike the primary training dataset, the real distribution dataset closely mirrors the natural class imbalances encountered in clinical practice, containing only 54 left-dominant and 346 right-dominant cases, along with significant variability in image quality, artifacts, and diagnostic uncertainty. These features make the real distribution dataset a rigorous benchmark for testing model robustness and clinical applicability.

The detailed classification performance metrics for all models on the real distribution dataset are shown in Table 8. Among the three models, VideoMAEv2 demonstrated the most robust and balanced performance. Achieving the highest accuracy (90.48%), it also secured the best F1-score (67.14%) and Matthews Correlation Coefficient (MCC = 0.6438), indicating strong performance across both majority and minority classes. Notably, VideoMAEv2 excelled in recognizing the minority class (left dominance), achieving the highest recall at 54.35%, making it particularly valuable for clinical decision support systems, where identifying rare but clinically significant cases is crucial. Furthermore, its high precision (87.81%), lowest false positive rate (1.64%), and low false discovery rate (12.19%) underscore its capability to produce sensitive yet reliable predictions.

The Video Swin Transformer also delivered a competitive performance, particularly characterized by high precision (85.94%) and low false positive rates, indicating fewer incorrect predictions of left dominance. However, its recall (48.76%) was somewhat lower than that of VideoMAEv2, implying a slight tendency to miss some left-dominant cases. Despite this limitation, its F1-score (62.22%) and MCC (0.5914) clearly outperform the TSN, positioning it as a cautious yet robust alternative.

In contrast, the TSN, while achieving a reasonable accuracy (88.37%), underperformed significantly in detecting the minority class. The TSN exhibited the lowest recall (48.10%), F1-score (54.72%), and MCC (0.488), alongside a notably high false discovery rate (36.56%). Although the TSN accurately classified the dominant (right dominance) class, its weakness in identifying left-dominant patterns severely limits its suitability in imbalanced and uncertain diagnostic scenarios.

For comparison purposes, the multi-stage classification approach of Kruzhilov et al. [9] on the same dataset reported superior outcomes in all primary metrics: accuracy (97.30%), precision (93.10%), recall (94.65%), F1-score (91.90%), and MCC (0.878). These results reflect the benefits provided by artery-specific modeling and extensive preprocessing steps; separating RCA and LCA images proved to be a principal component of this success. Although their model achieved remarkable recall and overall accuracy, its reliance on multiple condition-based decision branches and manually tailored processing steps might limit its scalability and real-time clinical integration potential.

Collectively, these results underscore the superior generalization capabilities of VideoMAEv2, likely attributable to its advanced pretraining strategies leveraging large and diverse video datasets, as well as its innovative dual-masking architecture. Among the evaluated models, VideoMAEv2 stands out as the most suitable candidate for clinical deployment, effectively and reliably identifying both common and rare classes in real-world angiographic data.

**Figure 12:**
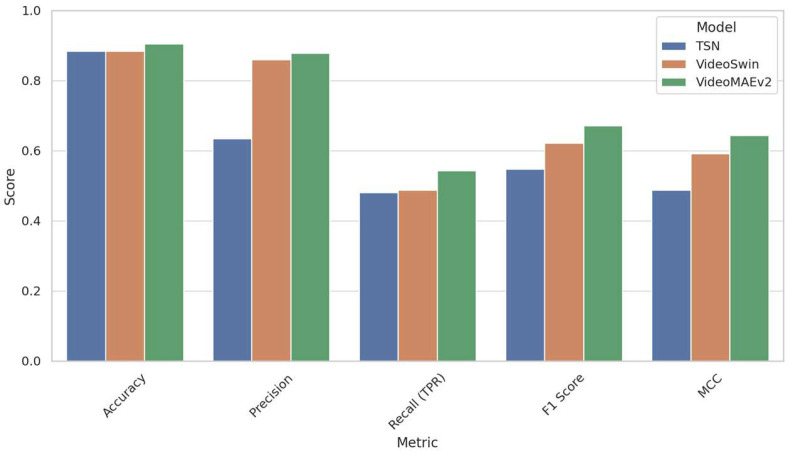
Comparative visualization of key performance metrics for TSN, Video Swin Transformer, and VideoMAEv2 on real distribution dataset.

Figure 12 provides a comparative visualization of the performance metrics of the three models—the TSN, Video Swin Transformer, and VideoMAEv2—evaluated on the real distribution dataset (RDS). Among these models, VideoMAEv2 consistently outperformed the others across key metrics including accuracy, precision, recall, F1-score, and Matthews Correlation Coefficient (MCC). This superior performance highlights VideoMAEv2’s robustness against the inherent challenges of real-world clinical data, such as class imbalance and variable image quality. In contrast, the TSN, despite achieving comparable accuracy, demonstrated significantly lower sensitivity and MCC scores. These shortcomings underscore TSN’s inadequacy in correctly identifying cases belonging to the minority (left-dominant) class. Meanwhile, the Video Swin Transformer showed a balanced yet conservative approach; although it achieved a high precision rate, its recall was somewhat lower compared to that of VideoMAEv2. Overall, these results reinforce the significance of advanced architectural designs and pre-training strategies—as exemplified by VideoMAEv2—in ensuring reliable generalization to heterogeneous clinical environments.

## 4. Discussion

### 4.1. Comparative Performance Analysis

The TSN model stands out due to its high computational efficiency and rapid inference time. Although achieving a satisfactory accuracy of 87.86%, its simple CNN-based architecture was less effective in capturing the intricate spatial and temporal variations inherent in angiographic videos. Leveraging its hierarchical, attention-based architecture, the Video Swin Transformer effectively modeled spatiotemporal localities, achieving an accuracy of 92.12%. However, this performance came at the cost of increased computational complexity compared to the TSN. Reaching the highest accuracy of 92.89%, VideoMAEv2 excelled through its dual-masking strategy and self-supervised learning paradigm. Its ability to capture richer feature representations while maintaining computational efficiency demonstrated its strong potential for integration into real-time clinical scenarios.

The extensive comparative analysis highlights each model’s unique strengths and limitations clearly. The TSN is computationally efficient and rapid, but struggles significantly with minority classes and precision-sensitive scenarios. The Video Swin Transformer provides exceptional precision, specificity, and overall stability, ideal for clinical decision support systems where reducing false positives is critical. VideoMAEv2 balances accuracy, sensitivity, and robustness with class imbalance, making it well suited for diverse clinical applications where labeled data may be limited and anatomical variation extensive.

One of the main reasons that transformer-based models outperform Convolutional Neural Networks (CNNs) lies in their effective modeling of long-range spatiotemporal dependencies. CNN architectures are typically limited by fixed-size receptive fields and primarily capture local features through layered convolutional structures [58]. This inherently limits their ability to effectively identify subtle anatomical or temporal cues spread over long frame intervals, representing a significant drawback in complex imaging tasks such as coronary angiography [26]. On the other hand, transformer architectures, including VideoMAEv2 and the Video Swin Transformer, utilize self-attention mechanisms that dynamically focus on spatially and temporally distributed regions of interest [58]. These advantages can be crucial for clinical decisions, where the precise analysis of vessel flow continuity and contrast agent propagation is essential [59].

Transformer models integrate spatial and temporal information holistically, unlike many CNN architectures that process such data in separate stages. This integration facilitates a more interpretable decision-making process, enabling cardiologists to operate more effectively in real-world clinical settings [60]. For instance, the masked autoencoder pre-training strategies employed in VideoMAEv2 allow for robust and semantically rich feature extraction, even in domains where large annotated datasets are limited [61]. This capability is particularly important in fields like coronary angiography, which frequently exhibits significant anatomical variability [26].

Comprehensive comparative analyses further highlight distinct the strengths and weaknesses across models. Temporal Segment Networks (TSNs) achieve rapid processing speeds, but frequently suffer from performance drops, especially for minority classes [60]. Conversely, the Video Swin Transformer provides high specificity and precision, making it particularly suitable for decision support systems where false positives can lead to serious clinical consequences [62]. VideoMAEv2, meanwhile, demonstrates resilience to class imbalance and emerges as a leading choice in clinical scenarios with substantial anatomical variability [26,59].

Although the highest-performing model in this study, VideoMAEv2, demonstrated promising results with 90.48% accuracy and 87.81% precision, it still remains below the performance levels reported by Kruzhilov et al. [9] on the same dataset, namely 97.30% accuracy and 93.10% precision. The most noticeable discrepancy lies in the recall (54.35% versus 94.65%) and F1-score (67.14% versus 91.90%), revealing that the model struggles especially in detecting minority-class instances (for example, left-dominant cases). Several fundamental factors may account for this difference: in this study, no distinction was made between RCA and LCA, and a single, source-independent model was employed. Furthermore, no vascular segmentation, artifact or low-quality frame removal, or artery-focused preprocessing steps were applied. Class imbalance was addressed solely through general training strategies, without the use of any specific balancing techniques. Even so, the model exhibits reliability by preventing the misclassification of the right-dominant class to a significant extent, as evidenced by its low false positive rate (FPR: 1.64%) and high true negative rate (TNR: 98.36%). These findings indicate an important trade-off between sensitivity and specificity, suggesting that incorporating targeted preprocessing steps or specialized strategies for class balance in future research may further enhance sensitivity and overall clinical utility.

These outcomes emphasize that deep learning-based video architectures are effective tools for coronary artery dominance classification. Yet, selecting the most appropriate model must carefully reflect specific clinical contexts and performance requirements.

### 4.2. Clinical Impact and Practical Implications

The integrated approach proposed in this study significantly simplifies the classification pipeline by eliminating the necessity for differentiating RCA/LCA origins. The robustness and high accuracy of the proposed model strongly support its adoption in clinical practice. Particularly, it offers a reliable, objective, and automated method for evaluating coronary dominance in routine angiographic assessments.

The key clinical and practical implications of the proposed approach include the following:

- Reduction in Observer Variability and Diagnostic Subjectivity: The automated classification system substantially reduces inter-observer variability and the subjective biases associated with manual dominance assessments, thus improving the consistency and reproducibility of clinical diagnoses;
- Seamless Integration with Clinical Picture Archiving and Communication Systems (PACSs): The model provides the potential for seamless integration into clinical PACSs, facilitating instant analyses during angiography. This immediate availability enables cardiologists to access real-time decision support, enhancing clinical workflow and patient management efficiency;
- Elimination of Preprocessing Requirements: Unlike previous methods requiring preprocessing steps such as vessel segmentation, image quality filtering, or RCA/LCA source differentiation, the proposed method operates directly on raw angiographic video sequences. By eliminating the need for source-specific preprocessing, this framework significantly simplifies the implementation, removing the dependency on multi-stage modeling pipelines or additional software modules. The capability to holistically process angiographic images independently of arterial origin substantially enhances clinical applicability and interpretability, particularly in complex or ambiguous cases such as co-dominance or vessel occlusion;
- Computational Efficiency and Real-Time Applicability: The proposed end-to-end architecture performs classification tasks with minimal latency and computational load, making it highly suitable for real-time integration into catheterization laboratories or emergency clinical settings. Its ability to directly infer classifications from full video inputs grants it a considerable advantage in both speed and scalability compared to frame-based or multimodal approaches. This real-time capability ensures that the method is practical and efficient enough for immediate clinical decision-making;
- Model-specific Clinical Recommendations: the TSN is suitable for deployment in rapid and resource-constrained environments due to its computational efficiency and short inference time. However, the relatively high misclassification rate for the minority left-dominant class might introduce risks in scenarios where the accurate identification of such minority groups is clinically critical. The Video Swin Transformer demonstrates exceptional specificity and precision and low false positive rates, offering reliable classification performance. This high precision is particularly beneficial in scenarios where false positives entail significant clinical risks or intervention costs. Nonetheless, inference speed constraints may limit its feasibility in strictly time-sensitive clinical settings. VideoMAEv2 delivers the most balanced solution in terms of sensitivity, overall accuracy, and minority class stability. Its robustness against data variability, high generalization capability, and interpretability makes VideoMAEv2 particularly suitable for clinical environments where comprehensive diagnostic accuracy, speed, and data flexibility are essential.

So, this integrated approach to coronary artery dominance classification not only improves accuracy and objectivity, but also aligns closely with the practical needs of clinical decision-making processes, potentially advancing routine angiographic practices toward more efficient, precise, and consistent standards.

## 5. Conclusions

This study proposes an integrated system aimed at automatically classifying coronary artery dominance from coronary angiographic video sequences, thereby filling an important gap in the existing literature. A video-based independent classification framework has been introduced, eliminating the need for predefined assumptions about whether the artery originates from the right or left system. Thus, compared to the current models based on RCA/LCA distinction, a simpler, more flexible, and more generalizable approach has been developed.

As a result of comprehensive comparisons, the Video Swin Transformer model has been distinguished for achieving the highest accuracy (96.2%) and MCC value (0.8533), whereas the VideoMAEv2 has drawn attention due to its resilience to class imbalances, ability to learn from unlabeled data, and more balanced performance. The TSN model, on the other hand, offers a preferable solution for time-constrained clinical applications due to its low computational cost and rapid inference time. Therefore, the potential of all three models has been identified, allowing for optimization based on differing clinical needs and available system resources.

One of the most significant contributions of this study is presenting an evaluation framework that is not limited to mere technical accuracy, but that also analyzes the clinical integration suitability of the models through multidimensional metrics. The combined evaluation of metrics such as F1-score, precision–recall balance, inference time, interpretability, data flexibility, and MCC provides important insights into the real-world applicability of artificial intelligence systems.

### Future Directions

The findings obtained from this study indicate that video-based deep learning systems can be integrated with clinical decision support systems in cardiology. However, for these technologies to enter routine clinical use, in-depth studies in the following research areas are recommended:

- The utilization of methods such as attention maps and Grad-CAM, which clearly demonstrate the video segments or vascular structures influencing the model’s decisions, will enhance clinical trust for explainability and visualization;
- The current models should be tested on extensive datasets obtained from diverse geographic regions, devices, and patient populations for prospective and multi-center clinical validation. This is crucial for verifying generalizability and reliability;
- Diagnostic accuracy could be improved by integrating other modalities such as electrocardiogram (ECG), echocardiogram (ECHO), clinical notes, and laboratory data in addition to angiographic videos via multimodality and multiple data sources;
- Hybrid structures combining the advantages of the Video Swin Transformer and VideoMAEv2 could yield more balanced solutions in terms of both accuracy and inference speed;
- Software engineering-level applications should be developed for integrating the models into existing imaging systems such as Picture Archiving and Communication Systems (PACSs) or Coronary Computed Tomography Angiography (CCTA) for integration into clinical workflow;
- User-friendly interfaces could be developed by designing explainable artificial intelligence solutions that support but do not share the responsibility for the final decisions of clinicians with expert–AI collaboration models.

Studies to be conducted in these directions will not just remain limited to coronary dominance classification, but also pave the way for fully integrated and autonomous artificial intelligence systems in the field of cardiovascular imaging. Ultimately, this study demonstrates the feasibility of AI-supported automation in cardiovascular diagnosis by presenting a video-based classification framework that is clinically realistic, generalizable, computationally efficient, and interpretable.

## Figures and Tables

**Table 1:** Distribution of dataset subsets.

Subset	Number of Samples	Percentage	Left Dominance	Right Dominance
Training	3610	70%	1102	2508
Validation	774	15%	236	538
Testing	773	15%	235	538
Total	5157	100%	1573	3584

**Table 2:** Model types, backbone architectures, and input configuration parameters.

Model	Model Type	Backbone	Input Format	Clip Length	Frame Interval
TSN	Recognizer2D	ResNet-50	NCHW	3 clips × 1 frame	-
VideoMAEv2	Recognizer3D	ViT-Base	NCTHW	1 clip × 16 frames	4
Video Swin Transformer	Recognizer3D	Swin Transformer 3D Large	NCTHW	1 clip × 32 frames	2

**Table 3:** Training- and testing-phase parameters for each model architecture.

Model	Batch Size	Optimizer	Learning Rate	Weight Decay	Scheduler	Pretrained Weights	Test Clips
TSN	32	SGD- momentum = 0.9	0.005	0.0001	MultiStepLR milestones: 20, 40	ResNet-50 (ImageNet)	25
VideoMAEv2	4	AdamW	0.001	0.05	LinearLR + CosineAnnealingLR	ViT-G (Kinetics-710)	5
Video Swin Transformer	2	AdamW	0.001	0.05	LinearLR + CosineAnnealingLR	Swin-L (ImageNet22K)	4

**Table 4:** Detailed classification performance metrics for all evaluated models.

Algorithm	Accuracy	Precision	Recall	F1-Score	TPR *	FNR *	FPR *	TNR *	FDR *	MCC *
TSN	90.31%	76.27%	90.45%	82.76%	90.45%	9.55%	9.74%	90.26%	23.73%	0.7662
Video Swin Transformer	93.54%	94.07%	86.05%	89.88%	86.05%	13.95%	2.71%	97.29%	5.93%	0.8533
VideoMAEv2	93.80%	88.98%	90.52%	89.74%	90.52%	9.48%	4.80%	95.20%	11.02%	0.8530

* TPR: true positive rate, FNR: false negative rate, FPR: false positive rate, TNR: true negative rate, FDR: false discovery rate, and MCC: Matthews Correlation Coefficient.

**Table 5:** Inference time and efficiency.

Model	Overall Accuracy	Inference Time (ms/frame)	Params (M)
TSN	91.96%	9.7	23
Video Swin Transformer	93.62%	14.1	88
VideoMAEv2	94.70%	12.3	96

**Table 6:** Comparative results for coronary dominance classification.

Model	Training Accuracy	Validation Accuracy	Test Accuracy
TSN	97.70%	90.31%	87.86%
Video Swin Transformer	95.21%	93.54%	92.12%
VideoMAEv2	96.65%	93.80%	92.89%

**Table 7:** Comparison of AUC values across training, validation, and test sets for each model.

Model	AUC (Training)	AUC (Validation)	AUC (Test)
TSN	0.9971	0.9367	0.9436
Video Swin Transformer	0.9836	0.9651	0.9666
VideoMAEv2	0.9936	0.9834	0.9781

**Table 8:** Detailed classification performance metrics for all models on real distribution dataset.

Model	Accuracy	Precision	Recall	F1-Score	MCC	TPR	FPR	TNR	FDR
TSN	88.37%	63.44%	48.10%	54.72%	0.488	48.10%	4.74%	95.26%	36.56%
VideoSwin	88.44%	85.94%	48.76%	62.22%	0.591	48.76%	1.93%	98.07%	14.06%
VideoMAEv2	90.48%	87.81%	54.35%	67.14%	0.643	54.35%	1.64%	98.36%	12.19%
Kruzhilov et al. [9]	97.30%	93.10%	94.64%	91.90%	0.878	-	-	-	-

## Data Availability

The data presented in this study are available in CoronaryDominance at https://huggingface.co/datasets/BearSubj13/CoronaryDominance (accessed on 6 March 2025), reference number [9].

## Abbreviations

The following abbreviations are used in this manuscript:

ACS	Acute coronary syndrome
AI	Artificial intelligence
AUC	Area under curve
CAD	Coronary artery disease
CCTA	Coronary Computed Tomography Angiography
CNNs	Convolutional Neural Networks
ECG	Electrocardiogram
ECHO	Echocardiogram
FDR	False discovery rate
FNR	False negative rate
FPR	False positive rate
GPU	Graphical Processing Unit
LCA	Left coronary artery
LCx	Left circumflex
LD	Left dominance
MAE	Masked autoencoder
MCC	Matthews Correlation Coefficient
PACSs	Picture Archiving and Communication Systems
PDA	Posterior descending artery
RCA	Right coronary artery
RD	Right dominance
TNR	True negative rate (specificity)
TPR	True positive rate (sensitivity)
TSN	Temporal Segment Network
VideoMAE	Video Masked AutoEncoder
VST	Video Swin Transformer
